# Pertussis in The Netherlands: an outbreak despite high levels of immunization with whole-cell vaccine.

**DOI:** 10.3201/eid0302.970211

**Published:** 1997

**Authors:** H E de Melker, M A Conyn-van Spaendonck, H C Rümke, J K van Wijngaarden, F R Mooi, J F Schellekens

**Affiliations:** National Institute of Public Health and the Environment, Bilthoven, The Netherlands.

## Abstract

In 1996, a sudden increase in pertussis incidence was reported in the Netherlands (2.1 per 100,000 in 1995, 18 per 100,000 in 1996). Although not all potential surveillance artifacts could be excluded, it is highly probable that the data reflect a true outbreak. However, the cause of this increase has not yet been determined. Further research is directed to the severity of disease and a possible mismatch between the vaccine and the circulating Bordetella strains.


### Pertussis in the Netherlands: an Outbreak Despite High Levels of Immunization with Whole-Cell Vaccine

In 1996, a sudden increase in pertussis incidence was reported in the Netherlands (2.1 per 100,000 in 1995, 18 per 100,000 in 1996). Although not all potential surveillance artifacts could be excluded, it is highly probable that the data reflect a true outbreak. However, the cause of this increase has not yet been determined. Further research is directed to the severity of disease and a possible mismatch between the vaccine and the circulating Bordetella strains.

In 1996, 2,771 cases of pertussis were reported to the Inspectorate of Health in the Netherlands (population 15 million), compared with 319 cases in 1995. With epidemic cycles expected every 3 to 5 years and a recent outbreak in 1994, this rise was unexpected (1). After the introduction of pertussis immunization with a whole-cell vaccine in the National Immunization Program (1952), the incidence of pertussis in the Netherlands decreased significantly. Children are immunized at ages 3, 4, 5, and 11 months with a diphtheria, tetanus, pertussis, and inactivated polio vaccine (DTP-IPV), and vaccine coverage for pertussis, for at least three immunizations, is 96% at the age of 12 months. Until the 1980s, the incidence of pertussis seemed very low, because only incidental cases were reported. However, in the last two decades, pertussis has been endemic, and epidemic peaks have appeared.

The surveillance of pertussis is based mainly on notification, which was made obligatory by law in 1976. Because the availability and interpretation of serologic tests changed in the 1980s and the absence of a clear case definition for notification seemed to have influenced surveillance, a restrictive case definition that included criteria for laboratory diagnosis was introduced in 1988. Therefore, we have limited this report to notification data from 1989 onwards. The annual incidence of pertussis notification from 1989 to 1995 is shown in Figure 1. In this period, the case definition includes defined clinical symptoms and laboratory confirmation. The clinical symptoms are a serious cough lasting more than 2 weeks or cough attacks or cough followed by vomiting in combination with at least one of the following symptoms/findings: apnea, cyanosis, characteristic cough with whooping, subconjunctival bleeding, leukocytosis, or contact with a person with confirmed or suspected pertussis in the previous 3 weeks. Laboratory confirmation is defined as either positive culture of *Bordetella pertussis* or *Bordetella parapertussis* or positive two-point serology, i.e., the finding of a significant rise (> 4-fold) of IgG antibodies against pertussis toxin and/or IgA antibodies against *B. pertussis* in paired sera. The case definition is highly specific, but results in significant underreporting.

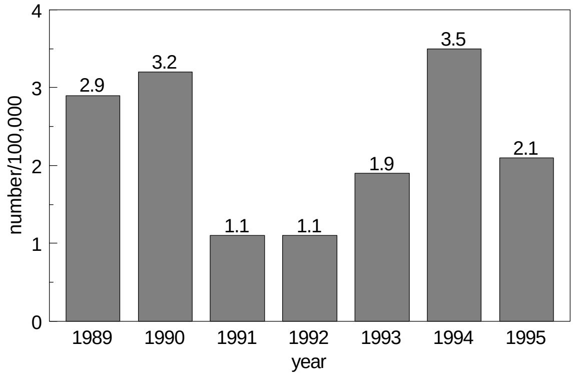

Figure 1. Annual incidence of pertussis estimated from notification by registration date, 1989–1995

We compared the number of cases reported per 4-week period in 1996 with the number reported in 1994, the year with the highest incidence in recent years (Figure 2). It appears that an unexpectedly large number of pertussis cases was reported (18 per 100,000) in 1996. The reports came from all over the country. No geographic clustering was observed, even in regions with pockets of low vaccination coverage or at the borders of

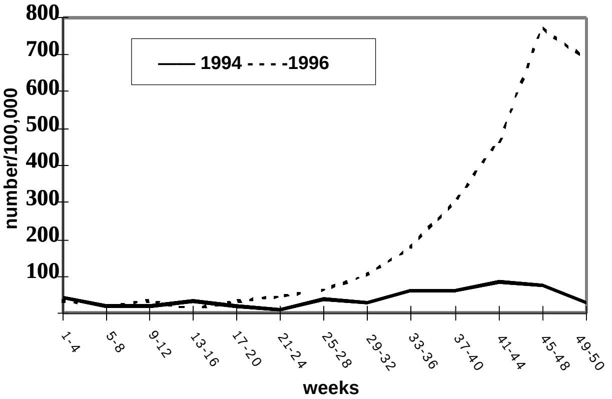

Figure 2. Number of cases reported by registration date, 1994 and 1996 (4-week periods).

the country (close to Germany, where vaccination coverage for pertussis is low).

The age distribution of patients in 1989 to 1996 shows a significant decrease in the proportion of infants less than 1 year old (from 21% in 1989 to 7% in 1996) and a significant increase in the proportion of 1- to 4-year-old children (from 21% in 1989 to 30% in 1996). A significant decrease (from 42% in 1989 to 30% in 1995) was also observed in 5- to 9-year-old children; however, in 1996, the proportion increased to 39%. No changes were observed in the 10- to 14- and 15- to 19-year-old groups, whereas the proportion of patients 20 years old or older increased from 5% to 11% (Table).

Table. Age distribution (percentages) pertussis notification in 1989 to 1996

| Age | 1989 | 1990 | 1991 | 1992 | 1993 | 1994 | 1995 | 1996 |
|---|---|---|---|---|---|---|---|---|
| 0 | 21 | 21 | 23 | 21 | 24 | 16 | 13 | 7 |
| 1-4 | 21 | 21 | 26 | 19 | 27 | 34 | 33 | 30 |
| 5-9 | 42 | 37 | 34 | 30 | 26 | 32 | 30 | 39 |
| 10-14 | 10 | 9 | 10 | 17 | 12 | 8 | 10 | 11 |
| 15-19 | 1 | 2 | 2 | 2 | 1 | 1 | 1 | 2 |
| ≥20 | 5 | 9 | 6 | 11 | 10 | 8 | 12 | 11 |
| Unknown | 0 | 0 | 1 | 1 | 0 | 1 | 1 | 0 |
| Total (n) | 434 | 471 | 164 | 169 | 294 | 536 | 319 | 2,771 |

Whereas the average vaccine coverage did not change, the proportion of vaccinated patients increased from 55% in 1989 to 85% in 1996. In all age groups, except that of 6- to 11-month old infants, the proportion of vaccinated patients is higher in the years 1994 to 1996 than in 1989 to 1993.

We estimated the annual incidence of pertussis for vaccinated and unvaccinated children ages 1 to 4 and 5 to 9 years, on the basis of reporting by registration date (1989 to 1996) (Figure 3). Calculation of incidence according to vaccination status was deliberately restricted to ages 1 to 4 years and 5 to 9 years (vaccine coverage estimated at 96%) for methodologic reasons. The changes in age-specific incidence over the years for vaccinated and unvaccinated children are identical for 1989 to 1993. However, from 1994 to 1996, and particularly in 1996, the incidence among vaccinated 1- to 4-year-olds was considerably higher than in 1989 and 1990, the years with the highest incidence in 1989 to 1993. In unvaccinated children, the incidence in 1994 and 1995 was lower than in 1989 and 1990. The difference in incidence between the period 1989 to 1990 and the year 1996 was much smaller in unvaccinated than in vaccinated persons. A similar discrepancy can be seen in the 5- to 9-year-old group.

In view of the increase in pertussis reporting in 1996, the first issue to be addressed was whether this reporting reflected a true increase in pertussis incidence or was a surveillance artifact. If considerable underreporting is assumed, an increase due to improved compliance to the notification system or increased alertness should be considered. However, in the first two quarters of 1996, there is no apparent reason for such an assumption. Nonetheless, after the first reports of the observed rise, both publicity and increased awareness of the disease by physicians and patients might have influenced reporting.

Serodiagnosis for the country as a whole is done by a single reference laboratory at the National Institute of Public Health and the Environment; thus, the data provided by the laboratory can be used for nationwide surveillance. Furthermore, the reference laboratory collects strains of *B. pertussis* from regional public health laboratories, and the number of strains received also reflects the epidemic curve. The epidemiologic curve derived from these sources over the past years is consistent with the notification data.

Since laboratory confirmation is part of the case definition, changes in serodiagnostic practice might have influenced notification. Frequently, no conclusive results are obtained from serology: a second serum sample may not be submitted, or the first serum sample may be taken late after onset of disease, thereby missing the dynamic phase of the immunoresponse. Recently, cut-off values for antibody titers characteristic for the acute immunoresponse after natural infection with *B. pertussis* have been determined by comparing the titer distribution in the healthy population,

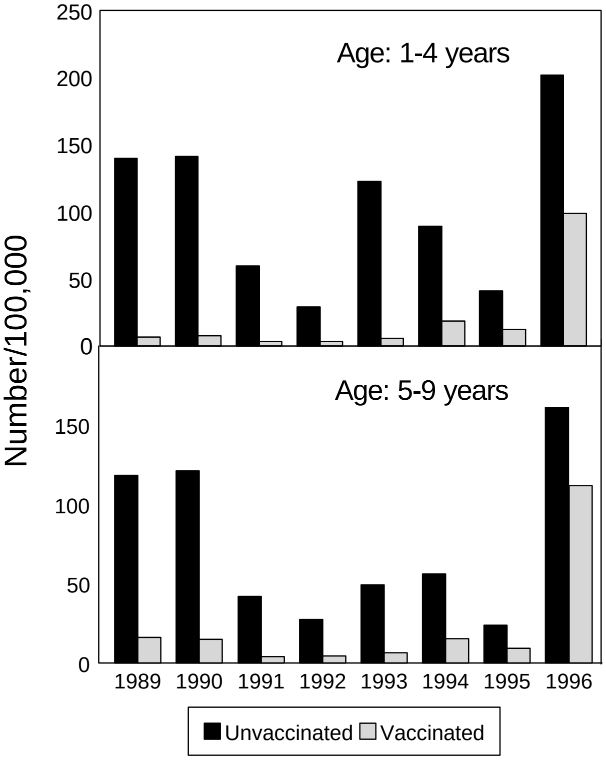

Figure 3. Estimated incidence of pertussis for unvaccinated and vaccinated children aged 1-4 years and 5-9 years based on notification records and assuming a coverage of 96% for the entire population.

in recently vaccinated children, and in sera longitudinally collected after natural infection. Since 1993, high titers in acute-phase sera are reported to be "suggestive for recent infection with *Bordetella pertussis*" (2). It can be imagined that such cases are reported, albeit not strictly in accordance with the case definition.

To exclude the possibility that the rise in notification was the result of reporting cases diagnosed with positive one-point serology only, we linked the notification and serodiagnosis databases. (The data were taken from January 1993 to September 1996.) Special permission to link the databases was given by the Inspectorate of Health. It appeared that some notification was based on positive one-point serology as early as 1993, and this proportion increased from 20% in 1993 to 33% in 1994. It remained at this level in the subsequent years. Thus, the 1996 increase in notification cannot be attributed to this change in serodiagnostic practice.

Our surveillance data suggest a decrease in vaccine efficacy, but estimation of vaccine efficacy from surveillance data should be interpreted with caution. However, there are no indications of significant bias from physicians' perceptions of vaccine efficacy that could have caused selective reporting of vaccinated patients; a higher probability of a positive serologic test result due to priming in vaccinated persons; or misclassification of cases with respect to vaccination status.

Although not all potential surveillance artifacts could be excluded, it is highly probable that the surveillance data reflect a true pertussis outbreak. The outbreak could have been caused by 1) decrease in vaccination coverage, 2) decrease in vaccine quality, 3) interference with other vaccines, 4) changes in circulating strains of *B. pertussis* that are not covered by vaccine-induced immunity, or 5) a combination of these factors. A decrease in vaccination coverage has not been observed in the (accurate) reporting system. The whole-cell vaccine used was produced in the National Institute of Public Health and the Environment and meets international standards. There was no sign of a gradual deterioration of vaccine quality as determined for product release by the mouse protection test.

In April 1993, vaccination against *Haemophilus influenzae* type b (Hib) was added to the immunization program. The polyribosylribitolphosphate tetanus toxoid conjugated vaccine (PRP-T) was administered simultaneously with the DTP-IPV, although on different limbs. Interference of Hib vaccination with the immunoresponse to pertussis vaccine has been described (3). In a prospective serologic study on the potential interference in the Netherlands, this effect was not observed (4). Furthermore, the increase of pertussis in 5- to 9-year-old vaccinated children, who never received Hib vaccine, was similar to the increase in the 1- to 4-year-old age group (Figure 3). Decreased vaccine efficacy due to interference of Hib vaccination is, therefore, very unlikely.

Molecular typing of *B. pertussis* isolates collected since 1945 indicated a shift in population structure, possibly due to the introduction of whole-cell vaccine (5). Further, antigenic variants of *B. pertussis* distinct from the strains incorporated in the vaccine appear to have emerged (6). It might be possible that circulating strains of *B. pertussis* have become less sensitive to vaccine-induced immunity. The abrupt increase in the proportion of vaccinated patients reported in all age groups suggests such a change.

Data from other countries in Europe indicate that the pertussis epidemic is restricted to the Netherlands. However, a resurgence of pertussis has been noted in the United States since the late

1980s and in Canada since 1991. No single factor has been found to explain this resurgence (7-10).

The decrease in pertussis notification in the Netherlands at the end of 1996 may indicate a seasonal variation in incidence. The 1996 data, including those on hospital admissions, are still being analyzed. These data will give insight into the severity of the pertussis epidemic among infants. Protection of these infants is the main reason for pertussis vaccination. In January 1997, active (monthly) surveillance by pediatricians of cases among hospitalized children was added to the routine surveillance based on notification and laboratory surveillance.

A prospective study is being considered to assess efficacy of the whole-cell vaccine, including differentiation of severity of illness and number of immunizations received, if the outbreak continues. Cooperative studies to determine the distribution of restriction fragment length polymorphism types and antigenic variants of *B. pertussis* in various countries in Europe, Asia, and North America are under way. Also, the immunogenicity of the Dutch whole-cell vaccine is being assessed: whether it has changed over time and how it compares to published immunogenicity data of whole-cell vaccines with known, prospectively determined, vaccine efficacy (11).

In conclusion, in the Netherlands a sudden increase of pertussis notification has been observed, which seems to reflect a true increase in incidence. Nevertheless, the cause of this increase has not been definitively determined. A possible mismatch between the vaccine and the circulating Bordetella strains is being investigated.

**H.E. de Melker,* M.A.E. Conyn-van Spaendonck,* H.C. Rümke,* J.K. van Wijngaarden,† F.R. Mooi,* and J.F.P. Schellekens***

*National Institute of Public Health and the Environment, Bilthoven, The Netherlands; and †Inspectorate of Health, Rijswijk, The Netherlands

### References

1. Melker HE de, Conyn-van Spaendonck MAE, Rümke HC, Sprenger MJW, Schellekens JFP. Kinkhoest in Nederland: 1989-1994. Nederlands Tijdschrift Geneeskunde 1995;139:1280-6.
2. Zee A van der, Agterberg C, Peeters MF, Mooi F, Schellekens JFP. A clinical validation of *B. (para)pertussis* PCR: comparison with culture and serology using samples of patients suspected for whooping cough from a highly immunized population. J Infect Dis 1996;174:89-96.
3. Clemens JD, Ferreccio C, Levine MM, Horwitz I, Rao MR, Edwards KM, et al. Impact of *Haemophilus influenzae* type b polysaccharide-tetanus-pertussis vaccine. JAMA 1992;267:880-4.
4. Labadie J, Sundermann LC, Rümke HC, and the DTP-IPV~Hib vaccine study group. Multi-center study on the simultaneous administration of DTP-IPV and Hib PRP-T vaccines. Part 1. Immunogenicity. National Institute of Public Health and the Environment (RIVM) Bilthoven, The Netherlands. Report No. 124001003, April 1996.
5. Zee A van der, Vernooij S, Peeters M, Embden J van, Mooi FR. Dynamics of the population structure of *Bordetella pertussis* as measured by IS1002-associated RFLP: comparison of pre- and post-vaccination strains and global distribution. Microbiology 1996;142:3479-85.
6. Mooi FR, Oirschot H van, Peeters J, Willems RJL. Antigenic variation of the accellular vaccine component pertactin in the Dutch *B. pertussis* population. National Institute of Public Health and the Environment (RIVM) Bilthoven, The Netherlands. Annual Report 1995.
7. Centers for Disease Control and Prevention. Resurgence of pertussis - United States, 1993. MMWR Morb Mortal Wkly Rep 1993;42:952-3,959-60.
8. Centers for Disease Control and Prevention. Pertussis - United States, January 1992-June 1995. MMWR Morb Mortal Wkly Rep 1995;44:525-9.
9. De Serres G, Bouliane N, Douville Fradet M, Duval B. Pertussis in Quebec: ongoing epidemic since the late 1980s. Can Commun Dis Rep 1995;21:45-8.
10. Milord F. Resurgence of pertussis in Monteregie, Quebec - 1990-1994. Can Commun Dis Rep 1995;21:40-4.
11. Greco D, Salmaso S, Mastrantonio P, Giuliano M, Tozzi AE, Anemona A, et al. A controlled trial of two acellular vaccines and one whole cell vaccine against pertussis. N Engl J Med 1996;334:341-8.
